# Characteristics and outcomes in a prospective cohort of patients with histologically diagnosed aortitis

**DOI:** 10.1093/rap/rky051

**Published:** 2019-01-25

**Authors:** Hart A Goldhar, Kyle M Walker, Mohamed Abdelrazek, Eric C Belanger, Munir Boodhwani, Nataliya Milman

**Affiliations:** 1 Department of Medicine, University of Ottawa, The Ottawa Hospital, Ottawa, Ontario, Canada; 2 Division of Rheumatology, University of Ottawa, The Ottawa Hospital, Ottawa, Ontario, Canada; 3 Department of Medical Imaging, University of Ottawa, The Ottawa Hospital, Ottawa, Ontario, Canada; 4 Department of Pathology and Laboratory Medicine, University of Ottawa, The Ottawa Hospital, Ottawa, Ontario, Canada; 5 Division of Cardiac Surgery, University of Ottawa Heart Institute, Ottawa, Ontario, Canada; 6 Department of Clinical Epidemiology, The Ottawa Hospital Research Institute, Ottawa, Ontario, Canada

**Keywords:** vasculitis, giant cell arteritis, temporal arteritis, other idiopathic inflammatory disorders, cardiovascular

## Abstract

**Objectives:**

Our aim was to evaluate characteristics and prospective adverse aortic outcomes in a cohort of patients with non-infectious histological aortitis.

**Methods:**

Patients with histological aortitis, diagnosed at the Ottawa Hospital after surgical repair of thoracic aortic aneurysms or dissections, consented to enrolment in a prospective observational cohort. Patients were assessed for an underlying inflammatory condition and followed prospectively with periodic clinical, laboratory and radiographic assessments. Aortic outcomes during follow-up included significant events, defined as new thoracic or abdominal aortic aneurysms, dissections, ruptures or other complications requiring aortic intervention, in addition to aortic branch ectasias, aneurysms and stenosis.

**Results:**

Sixteen patients with histological aortitis from surgical procedures performed between 2010 and 2017 were included; nine had idiopathic and seven had secondary aortitis. Idiopathic patients were more likely to have smoked (100 *vs* 43%, *P* = 0.02) and had more associated arch or descending aortic aneurysms on pre-operative baseline imaging compared with secondary aortitis (6 *vs* 0, *P* = 0.01). At the median 3.6 years of follow-up, eight patients (50%) had 10 significant aortic events. The incidence of aortic dissection was higher in the first year post-surgery, compared with subsequent years, whereas incident aneurysms occurred throughout follow-up. Elevated inflammatory markers during follow-up trended towards association with accumulation of severe aortic damage.

**Conclusion:**

This is the first reported prospective study in patients with histological aortitis. Within the limitations of a small cohort, we report a high incidence of aortic complications. Studies with a larger sample size and longer follow-up are needed to corroborate these findings.

**Key messages**

- Non-infectious aortitis carries a high incidence of subsequent adverse aortic events, including aneurysms and dissections.
- Incident aortic aneurysms developed throughout follow-up, whereas dissections occurred in the first post-operative year.

## Introduction

Aortitis is a group of disorders characterized by inflammation of the aorta. It is diagnosed either on histopathology, when active inflammatory infiltrates that involve the intima and/or media are demonstrated [1], or radiographically as circumferential aortic wall thickening on MRI or CT scanning, with or without corresponding increased fludeoxyglucose uptake on fludeoxyglucose PET scanning [2]. Aortitis is broadly classified into infectious (most commonly caused by bacterial pathogens) and non-infectious; the latter can be associated with a variety of inflammatory conditions [in which case, we refer to the aortitis as secondary aortitis (SA)] or be idiopathic (IA), designated when there are insufficient features to make a diagnosis of a defined inflammatory condition.

The term isolated aortitis was included in the Chapel-Hill classification under the category of single-organ vasculitis [3], and a more recent consensus statement on classification of aortitis by Stone *et al.* [1] proposed an alternative term, clinically isolated aortitis (CIA). Although the terms IA, CIA and isolated aortitis are frequently used interchangeably in published literature [2, 4–6], we use the term IA to refer to inflammation in any part of the aorta or its branches in the absence of an underlying defined inflammatory condition, and CIA to represent a subset of IA where disease is limited to the aorta and does not extend to its branches.

Idiopathic aortitis and CIA are of particular interest for a number of reasons. Firstly, at present IA and CIA are poorly defined, with data consisting entirely of small retrospective and case–control studies. Given that many cases of IA have features of a systemic large vessel vasculitis (but insufficient for firm diagnosis) and some IA patients in retrospective series end up being diagnosed with a defined inflammatory condition at follow-up, some sources suggest that cases of IA/CIA might be a limited expression of systemic large vessel vasculitides, such as GCA or Takayasu’s arteritis [3], or other inflammatory conditions [2]. Secondly, data suggest that these patients might be at highest risk for future aortic complications. In a retrospective surgical cohort from Massachusetts General Hospital, 15 IA patients were matched to 30 non-aortitis patients who also underwent ascending aortic aneurysm repair; strikingly, there was an 11-fold increased risk (*P* = 0.003) of aortic events in IA compared with the non-aortitis controls, throughout the 8-year follow-up [7]. In a French cohort of 117 cases of radiographically diagnosed aortitis, the rate of recurrent aortic events (aneurysm, ectasia, dissection, stenosis and/or surgery) was >2-fold higher in IA compared with GCA aortitis (54.5 *vs* 27.4%, *P* = 0.003) over a median follow-up of 34 months [8]. Lastly, the optimal management strategy of IA and CIA is unclear. Studies thus far have been mixed and of insufficient quality to indicate a benefit for immunosuppression or lack thereof [2, 8–11].

The purpose of this study was to use prospective observational data from a cohort of histologically diagnosed aortitis cases to characterize IA and SA with respect to baseline risk factors and clinical, biochemical, histological and radiological parameters; to evaluate the rate of aortic complications at follow-up; to determine predictors of aortic damage; and to ascertain whether, and to what degree, IA ultimately transforms into a systemic inflammatory condition.

## Methods

This study was conducted at the Ottawa Hospital (TOH), a network of three teaching hospitals affiliated with the University of Ottawa and the University of Ottawa Heart Institute (UOHI). The study was approved by the Ottawa Health Science Network Research Ethics Board. Informed consent was obtained from each patient at the time of enrolment into the study.

A collaboration was established in February 2013 between the Division of Rheumatology and the Department of Pathology and Laboratory Medicine at TOH and the Division of Cardiac Surgery at the UOHI with regard to subjects with histological evidence of aortitis. Histological aortitis is defined by our pathologist as active inflammation of the aortic wall that involves the media and/or intima; the inflammation can be of any histological pattern but should not be secondary to an infection or atherosclerosis. Upon identification of an adult patient who had surgical repair or resection of the aortic valve or thoracic aorta for any indication with aortic histology consistent with aortitis, additional biochemical and radiographic testing is ordered by the responsible cardiac surgeon. This includes ESR and CRP, testing of basic autoimmune markers, infectious work-up, including testing for syphilis and tuberculosis, and cross-sectional imaging of the whole length of the aorta and its branches, if not done before surgery. The cross-sectional imaging generally consists of CT angiography or, occasionally, abdominal Doppler ultrasound or MR angiography for patients who preferred to limit radiation exposure. The patient is then referred to our specialized vasculitis clinic.

At the initial visit in the vasculitis clinic, detailed clinical assessment is performed, including a review of demographic information, medications, atherosclerotic risk factors, features of rheumatological diseases, certain infectious diseases and other systemic illnesses, in addition to the available biochemical investigations and imaging tests. Any missing biochemical or radiographic studies are ordered, and temporal artery biopsy is performed for patients with a clinical suspicion of GCA.

Patients with histological evidence of aortitis determined to have non-infectious aortitis based on clinical assessment and investigations are invited to participate in our prospective cohort. At the minimum, consenting patients are seen at baseline, at 6 months and then yearly; each visit is associated with clinical assessment, measurement of ESR and CRP, and cross-sectional imaging of the thoracic aorta (CT angiography or MR angiography). Repeat imaging of the abdominal aorta and its branches is performed at 1–2 years post-operatively and then every 2–3 years. The frequency of follow-up visits and appropriate investigations is increased for patients with active disease or those felt to be at higher risk for complications. Treatment decisions are made on a case-by-case basis. Recruitment into the study started in September 2014 and is ongoing; this paper summarizes the cohort enrolled up to November 2017 and data collected to the end of December 2017.

Outcomes of interest at follow-up included the following: aortic significant events, defined as new thoracic or abdominal aneurysms, dissections, ruptures or other complications requiring intervention; other events of interest, namely branch ectasias, aneurysms and stenoses; and diagnosis of an associated inflammatory condition in patients with IA. Ectasia was defined as ≥4.0 cm for the thoracic and ≥3.0 cm for the abdominal aorta, and aneurysm as ≥5.0 and ≥4.0 cm respectively; a similar definition was used previously [7]. We also looked at predictors of cumulative aortitis-related damage, which was graded as mild, moderate or severe. We defined severe damage in the presence of an aortic dissection, rupture or endoleak; moderate damage in the presence of a distal aneurysm or other vascular lesions (ectasia, stesnosis) in at least three locations throughout the aorta; and mild damage in patients with vascular lesions not meeting these thresholds.

For the purpose of description of baseline parameters, we used biochemical tests performed upon discovery of histological aortitis or upon initial consultation with rheumatology; the exception was inflammatory markers (ESR and CRP), where the first measurement performed ≥3 months after surgery was considered baseline, to avoid false elevations related to the immediate post-surgical state. We used historical data and clinical findings obtained at the initial rheumatology consultations. For baseline imaging, pre-operative scans were used for the thoracic aorta, and the first available scan, which was generally performed between 3 and 6 months post-operatively, was used for the abdominal aorta.

Statistical analysis included descriptive statistics using Microsoft Excel, Fisher’s exact test [12] and the Mann–Whitney *U*-test [13] for significance. *P-*values were reported for baseline data but not for outcomes tables owing to the limitation of small sample size; a *P*-value of ≤0.05 was considered significant.

## Results

### Baseline information

Sixteen patients met inclusion criteria for histological aortitis (Table 1). The baseline clinical visit occurred within 9 months of the pertinent surgery for all patients. The mean age at diagnosis was 74 years. Nine patients had no associated inflammatory diagnoses (thus, IA), whereas seven had SA: five had GCA and two had RA. Of the seven SA patients, two received their associated diagnosis peri-operatively (one GCA, one RA), at the first rheumatology assessment, with the remaining five having a pre-existing secondary diagnosis. All patients tested negative for hepatitis, syphilis and tuberculosis. Only two patients were male, both with IA. All IA patients were remote or active smokers, compared with 43% of SA patients (*P* = 0.01). The indication for surgery was ascending aortic aneurysm in all but one SA patient, who had aortic dissection without aneurysm. Eleven of 16 (69%) patients had additional baseline aortic lesions outside the ascending aorta. Baseline aortic arch or descending aneurysms were seen only in the IA group (six aneurysms in four patients *vs* none in SA, *P* = 0.01). None of the patients had aortic wall thickening on baseline imaging. Eighty-one per cent of the cohort had giant cells on pathology, 56% had lymphocytic and plasma cells, and 44% had both; medial necrosis and fibrosis were seen in 87 and 80% of specimens, respectively. Pathological findings were comparable between groups (Table 1).

**Table 1:** Baseline demographic and medical information, aortic parameters and other laboratory results

	Idiopathic aortitis, *n =* 9	Secondary aortitis, *n *= 7	Full cohort *n* = 16	***P-*value**^a^
Female, *n* (%)	7 (78)	7 (100)	14 (88)	0.48
Age, years	72.9	74.3	73.5	0.27
Number of cardiovascular risk factors, mean	2.6	1.7	2.2	0.19
Smoker, *n* (%)	9 (100)	3 (43)	12 (75)	0.02
Diabetes, *n* (%)	0	1 (14)	1 (6)	0.44
Dyslipidaemia, *n* (%)	3 (33)	2 (29)	5 (31)	1.00
Hypertension, *n* (%)	6 (67)	2 (29)	8 (50)	0.31
Coronary artery disease, *n* (%)	2 (22)	0	2 (13)	0.48
Peripheral vascular disease, *n* (%)	1 (11)	1 (14)	2 (13)	1.00
Chronic kidney disease, *n* (%)	0	0	0	
Baseline medications				
Aspirin, *n* (%)	3 (33)	0	3 (19)	0.21
Other anti-thrombotic, *n* (%)	0	0	0	
ACEI or ARB, *n* (%)	3 (33)	3 (43)	6 (35)	1.00
Statin, *n* (%)	4 (44)	3 (43)	7 (44)	1.00
β-Blocker, *n* (%)	5 (56)	2 (29)	7 (44)	0.36
Prednisone, *n* (%)	1 (11)	0	1 (6)	1.00
Other immunosuppressant, *n* (%)	0	0	0	
Clinical features				
Aortic dissection, *n* (%)	0	1 (14)	1 (6)	0.44
Moderate-to-severe/severe aortic insufficiency, *n* (%)	5 (56)	2 (29)	7 (44)	0.36
Baseline imaging^b^				
Aortic root or ascending aorta aneurysm at site of surgery, *n* (%)^c^	9 (100)	6 (86)	15 (94)	0.44
Maximal diameter, mm, mean ±s.d.	59.3 ± 4.8	60.6 ± 6.2	59.8 ± 5.1	
Additional vascular lesions, total, *n*	19	10	29	
Aortic lesions, total, *n*	14	5	19	
Thoracic aorta, total, *n*	12	4	16	
Aortic arch ectasia, *n*	3	1	4	
Aortic arch aneurysm, *n*	3	0	3	
Descending aortic ectasia, *n*	3	3	6	
Descending aortic aneurysm, *n*	3	0	3	
Abdominal aortic aneurysms, *n*				
Suprarenal	1	1	2	
Infrarenal	1	0	1	
Branch lesions, total, *n*	5	5	10	
Branch ectasia/aneurysm, *n*	2	4	6	
Branch stenosis/occlusion, *n*	3	1	4	
Histopathology				
Giant cells, *n* (%)	8 (89)	5 (83)	13 (81)	0.55
Lymphoplasmacytic pattern, *n* (%)	5 (56)	4 (67)	9 (56)	1.00
Medial necrosis, *n* (%)	8 (89)	5 (83)	13 (87)	0.55
Medial degeneration, *n* (%)	3 (33)	3 (50)	6 (40)	0.62
Medial fibrosis, *n* (%)	8 (89)	4 (67)	12 (80)	0.52
Intimal fibrosis, *n* (%)	6 (67)	1 (17)	7 (47)	0.12
Adventitial fibrosis, *n* (%)	4 (44)	3 (50)	7 (47)	1.00
Atherosclerosis, *n* (%)	3 (33)	3 (50)	6 (40)	0.62
Initial inflammatory markers^d^				
ESR (mm/h)	21.1	16.3	18.7	0.80
CRP (mg/l)	6.8	19.5	12.7	0.35

a Statistical significance was calculated using Fisher’s exact test or the Mann–Whitney *U*-test; *P*-values of ≤0.05 were considered significant.

b Baseline aortic parameters were recorded from imaging pre-operatively; for those patients without abdominal imaging pre-operatively (*n* = 10), the first post-operative abdominal imaging was considered baseline (time from surgery to imaging ranged from 1 to 19 months)*.* One patient was initially lost to follow-up and thus had the first abdominal imaging after 84 months.

c *n* in this table refers to total lesions, rather than total patients. Fifteen of 16 patients had surgery for an aneurysm, and one patient had surgery for a dissection.

d Baseline values of inflammatory markers were those first recorded ≥90 days post-operatively, to exclude changes related to the post-surgical state. These values were recorded 3–14 months post-operatively. ACEI: angiotensin-converting enzyme inhibitors; ARB: angiotensin receptor blockers.

### Aortic events

Median follow-up was 3.6 (range 0.4–7.5) years, with an average of 4.6 imaging studies per patient, and 15 of 16 patients (94%) having at least two clinic visits and at least three imaging studies during follow-up. Eight patients in the cohort (50%) suffered significant aortic events: five patients in the IA group (56%) and three in the SA group (43%) (Table 2); this corresponds to seven and three total events in the respective groups. Types of events were similar between groups. Five patients developed new thoracoabdominal aneurysms, three IA and two SA. Aortic dissection occurred in three patients, all of which required surgical repair; one of these patients required a second intervention, for persistent endoleak. Dissections occurred only in the first post-operative year (after 4–8 months), whereas incident thoracoabdominal aneurysms were distributed throughout follow-up. The rate of growth of aortic dilatation was maximal in the first year after surgery (data not shown). Incident branch stenoses occurred in four IA patients and none of the SA patients.

**Table 2:** Cumulative number of patients with (and total number of) aortic events throughout duration of follow-up

	Idiopathic aortitis (*n = *9)	Secondary aortitis (*n =* 7)	Total (*n =* 16)	**Total events by year**^a^
Median follow-up, years	4.1 (0.4–7.5)	3.2 (1.8–6.5)	3.6 (0.4–7.5)	
New thoracic aortic aneurysm, *n*	3 (3)	2 (2)	5 (5)	1/2/2
New abdominal aortic aneurysm, *n*	1 (1)	0	1 (1)	0/0/1
Aortic dissection, *n*	2 (2)	1 (1)	3 (3)	3/0/0
Aortic rupture, *n*	0	0	0	
Other complication requiring intervention, *n*	1 (1)	0	1 (1)	0/1/0
New branch ectasia/aneurysm, *n*	2 (2)	2 (2)	4 (4)	2/1/1
New branch stenosis/occlusion, *n*	4 (4)	0	4 (4)	3/0/1
Significant aortic events^b^, *n*	5 (7)	3 (3)	8 (10)	4/3/3

a The numbers in the Total events by year column demonstrate whether the specified events occurred within the first year post-operatively/between 1 and 2 years post-operatively/or thereafter.

b Significant aortic events is a composite outcome corresponding to new thoracic or abdominal aortic aneurysms, dissection, ruptures or other complications requiring aortic intervention.

### Effect of medical therapy

Six patients received medical therapy remotely (all SA), and only 3 of 16 patients were actively treated during the study period, for cumulative 10.9 patient-years. One of these patients was treated for active RA rather than active aortitis; this patient received prednisone, MTX, plaquenil, leflunomide (all transiently), and then adalimumab followed by abatacept. Both patients treated for active aortitis had IA. The first patient was started on prednisone 4 months post-operatively after she developed a dissecting descending aortic aneurysm and ulcer requiring repair; later, she was also started on MTX and continued on prednisone owing to persistently elevated inflammatory markers. The second patient was started on prednisone (transiently) and MTX after a recurrent ascending aortic aneurysm, distal to the first surgical site, expanded from 50 to 55 mm, which subsequently stabilized over 18 months of follow-up. Within the limitations of these small group sizes, having received active or remote therapy did not affect the development of aortic events (Table 3). The two significant events that occurred in patients on therapy were an endovascular procedure for a pre-existing post-dissection endoleak that re-expanded, and insidious development of a suprarenal aortic aneurysm. None of the SA patients treated remotely required further treatment during the period of follow-up.

**Table 3:** Number of aortic events stratified by treatment status

Treatment status	Never treated	**Treated remotely**^a^	Actively treated	Total
Patients (patient-years)	9^b^ (27.9)	6 (19.7)	3^b^ (10.9)	18^b^ (58.5)
Idiopathic aortitis, *n* (%)	7 (78)	0	2 (22)	9
Secondary aortitis, *n* (%)	0	6 (86)	1 (14)	7
New thoracic aortic aneurysm, *n* (events per patient-year)	3 (0.11)	2 (0.10)	0	5 (0.09)
New abdominal aortic aneurysm, *n* (events per patient-year)	0	0	1 (0.09)	1 (0.02)
Aortic dissection, *n* (events per patient-year)	2 (0.07)	1 (0.05)	0	3 (0.05)
Further surgical aortic intervention, *n* (events per patient-year)	0	0	1 (0.09)	1 (0.02)
New branch ectasia/aneurysm, *n* (events per patient-year)	2 (0.07)	2 (0.10)	0	4 (0.07)
New branch stenosis/occlusion, *n* (events per patient-year)	4 (0.14)	0	0	4 (0.07)
Significant aortic events, *n* (events per patient-year)	5 (0.18)	3 (0.15)	2 (0.18)	10 (0.17)

a Remote treatment refers to having ceased immunosuppressive therapy ≥6 months before diagnosis of aortitis.

b Two patients had events before initiation of treatment, one of whom later had an event on treatment; they are represented in both groups.

### Prognostic factors

Four patients (25%) accumulated severe damage during follow-up, in all cases as a result of aortic dissection (Table 4). One of the four patients was treated remotely, and none actively. Both males in the cohort had mild disease. There was a trend towards increasing severity of damage with age at diagnosis and with the presence of baseline concomitant aortic arch and descending aorta aneurysms. The size of the initial ascending aneurysm was not correlated with future severity of damage. Three of four patients (75%) with severe damage had elevated mean ESR or CRP over the duration of follow-up, compared with 43% with moderate and 20% with mild damage. There was no difference in specificity between ESR and CRP, and no single patient had both markers elevated on average during follow-up.

**Table 4:** Predictors of severity of damage in aortitis

Severity of damage	Mild	Moderate	Severe
*n*	5	7	4
Median follow-up (years)	2.7	4.2	3.8
Female, *n* (%)	3 (60)	7 (100)	4 (100)
Mean age, years	71.6	73.7	75.5
Idiopathic, *n* (%)	4 (80)	3 (43)	2 (50)
Number of cardiovascular risk factors, mean	2.0	2.4	2.0
Smoker, *n* (%)	5 (100)	4 (57)	3 (75)
Diabetes, *n* (%)	0	1 (14)	0
Dyslipidaemia, *n* (%)	2 (40)	1 (14)	2 (50)
Hypertension, *n* (%)	3 (60)	4 (57)	3 (75)
Coronary artery disease, *n* (%)	0	1 (14)	1 (25)
Peripheral vascular disease, *n* (%)	0	1 (14)	1 (25)
Chronic kidney disease, *n* (%)	0	0	0
Baseline imaging			
Ascending aneurysm >6.0 cm, *n* (%)	3 (60)	3 (50)^a^	2 (67)^a^
Arch aneurysm, *n* (%)	0	1 (14)	2 (50)
Descending aneurysm, *n* (%)	0	1 (14)	2 (50)
Abdominal aneurysm, *n* (%)	0	1 (14)	1 (25)
Branch involvement, *n* (%)	0	2 (29)	2 (50)
Medications^b^			
Aspirin, *n* (%)	1 (20)	4 (57)	2 (50)
Other antithrombotic, *n* (%)	0	0	0
ACEI or ARB, *n* (%)	2 (40)	1 (14)	1 (25)
Statin, *n* (%)	3 (60)	4 (57)	3 (75)
β-Blocker, *n* (%)	2 (40)	5 (71)	3 (75)
Treatment status^b^			
Never treated, *n* (%)	4 (80)	2 (29)	3 (75)
Remotely treated, *n* (%)	1 (20)	4 (57)	1 (25)
Actively treated, *n* (%)	0	1 (14)	0
Inflammatory markers^c^			
Mean ESR >20 (mm/h)	1 (20)	2 (29)	1 (25)
Mean CRP >10 (mg/l)	0	1 (14)	2 (50)

a Baseline aortic diameters were missing for 2 of 16 patients and could not be extrapolated from post-operative imaging because grafts had already been placed.

b As recorded at the time of accumulation of that damage.

c The mean ESR and CRP for each patient during follow-up was calculated, beginning after 90 days post-operatively, to exclude the post-surgical state. The average number of laboratory measurements per patient was 8.3 (range 2–26), but two patients had none (follow-up period <90 days). ACEI: angiotensin-converting enzyme inhibitors; ARB: angiotensin receptor blockers.

## Discussion

This study describes a prospective cohort of 16 patients with histological aortitis, with a median follow-up of 3.6 years. Significant aortic events occurred in 8 of 16 patients. Dissections were more common in the first post-operative year, whereas new aneurysms occurred throughout follow-up. Within the limitations of a small cohort, medical therapy did not alter the incidence of events. Patients with baseline aortic arch and descending aneurysms trended towards accumulating more severe damage during follow-up. To our knowledge, this is the first prospective study evaluating outcomes in aortitis patients.

The mean age in our cohort, 74 years, was older than the range of 63–71 years in related retrospective studies [4, 9–11, 14–16]. Given that all our patients had surgery after 2010, this discrepancy might be explained by the trend towards increased longevity over time, improved surgical candidacy of elderly patients and improved surgical safety. The proportion of IA in our cohort, 56%, is in agreement with the retrospective studies, where IA tends to be the commonest aortitis, representing between 47 and 80% of the total cohorts [4, 6, 10, 11, 15]. The younger age and greater smoking prevalence observed among IA patients is also consistent with other studies [8, 17], as is the high prevalence of abnormalities outside the ascending aorta at baseline (69% in our study compared with 71–72% in two retrospective studies with total of 96 aortitis patients) [9, 10].

During follow-up, eight patients in our cohort developed significant aortic complications, including five patients (31%) with new aortic aneurysms. A number of earlier retrospective studies demonstrated similar significant rates of new aortic lesions, with rates of development of new aortic aneurysms ranging from 17% (6 of 36 patients) over a mean 3-year follow-up in a retrospective study from the Cleveland Clinic [11] to 42% (5 of 12 patients) over a mean of 47.5 months in the retrospective cohort study from our centre [10]. These high event rates also corroborate the 11-fold increased risk observed in an aforementioned retrospective study comparing IA patients with non-aortitis matched controls throughout 8 years of follow-up [7]. Likewise, in another study of 735 aortic surgery patients, the 5-year re-operation rate was 13% for aortitis, 6% for medial degeneration and 9% for atherosclerosis [16]; the re-operation rate in our cohort was 19%. In contrast, a 2006 study demonstrated relatively low rates of aortic aneurysm development in 21 IA patients [4], equivalent to 0.6% per year [2]. However, given that the proportion of subjects who had follow-up imaging was not specified, it is likely that that study described a different patient population; notably, six deaths were reported in the study, with no details regarding the cause.

Although the difference between IA and SA for patients with events was small (56 *vs* 43%), the difference for total events was more substantial (seven *vs* three). In the aforementioned radiographic aortitis cohort of 117 patients comparing IA with GCA patients, the aortic event rate was also markedly different at 55 *vs* 27%, respectively (*P* = 0.003), over 34 months [8]. Overall, although no definitive conclusions can be drawn in view of small sample sizes, a sizeable body of evidence seems to portray the alarming rate of recurrent aortic events in aortitis generally, and IA specifically.

Current evidence for a role of medical therapy in decreasing aortitis complications is mixed. In the present study, the evaluation of an effect of medical therapy was limited by small numbers and confounding by indication, because therapy was initiated in patients felt to be at high risk for complications. In the Cleveland Clinic aortitis cohort, 0 of 11 steroid-treated *vs* 6 of 25 untreated aortitis patients developed aneurysms during follow-up [11]. In the previously referenced radiological cohort of 44 IA patients, 40 were treated with steroids; of those without baseline aneurysms, 2 of 4 (50%) untreated *vs* 4 of 23 (17%) treated patients developed aneurysms [8]. However, a number of other studies did not demonstrate a similar signal of a treatment benefit [4, 9, 10]. Notably, steroids are not without risk in this population; a recent study of 176 patients with fusiform non-inflammatory abdominal aortic aneurysms demonstrated a significant correlation between rapid aortic expansion and oral steroid use for unrelated indications [18]. Further follow-up and larger samples are required to clarify the risk *vs* benefit of CSs in treating aortitis.

We observed that patients with more diffuse disease at baseline tended to have higher risks of accumulating moderate or severe damage. We made a similar observation previously in our retrospective study [10], whereby no patients with new aortic lesions during follow-up had disease confined to the ascending aorta at baseline. Additionally, we found that an elevated mean ESR or CRP over follow-up was associated with increased severity of aortic damage.

In our cohort of aortitis patients diagnosed on surgical specimens, there were no radiological features of aortitis, generally defined as ≥3 mm circumferential aortic wall thickening on CT or MRI [19]. This agrees with previous findings of rare wall thickening in cases of aortitis diagnosed histologically [20]. One possible explanation is that the thoracic aneurysms that upon resection reveal aortitis are a manifestation of a late stage of the disease, when the aortic wall thickening characteristic of earlier disease is largely resolved [21].

None of our nine IA patients developed a defined inflammatory condition during the median 4.1-year follow-up. Two of 32 IA patients in our previous retrospective study [10] were ultimately diagnosed with an inflammatory condition (cutaneous lupus and RA), occurring 6.2 and 8 years post-surgery. In another study of 48 patients with aortitis and GCA, only one patient had aortic disease diagnosed before GCA, occurring 5 months earlier [22]. Contrasting this is a finding of 23 of 73 (31.5%) IA patients developing a systemic inflammatory condition during follow-up in an unpublished report of a retrospective cohort [23]; the discrepancy might stem from ambiguity in defining criteria for diagnosis of the systemic conditions.

The major limitation of the present study is the small sample size and corresponding small event rates. Consequently, we felt that calculations of statistical significance would have been inappropriate, and we instead presented trends apparent to visual inspection of the data. Aortitis is rare, which makes prospective studies challenging and time consuming. However, in view of the lack of other prospective data on this group of conditions, we feel that our data nevertheless add significantly to the body of knowledge. Another limitation was that the imaging studies between and within patients were interpreted by different radiologists, and measurements could not be calibrated perfectly among them. This could possibly have led to millimetre differences in reporting diameters, although overall it is unlikely to have affected diagnosis of significant events (aneurysms or dissections). Other limitations include the lack of pre-operative inflammatory markers and pre-operative imaging of the abdominal aorta in most patients.

Strengths of the present study include the prospective design, which is unique among related literature. Furthermore, our patient population represents the complete sample of patients with histologically diagnosed aortitis at the Ottawa Hospital since the establishment of the multidisciplinary collaboration in February 2013, because all aortic specimens at our institution are processed by our collaborating pathologist, all patients with aortitis were seen in our vasculitis clinic and invited to participate in this study, and none declined participation. We also had a reasonably long median follow-up of 3.6 years, and the participants had nearly complete baseline and follow-up data, with an average of 4.6 follow-up imaging studies per patient.

In conclusion, we present here the first prospective cohort of 16 aortitis patients diagnosed on pathology after surgical aortic resection. Half of the patients developed significant aortic events after a median of 3.6 years, including three dissections, with a trend towards increased events in IA patients compared with SA. Dissections, but not incident aneurysms, occurred most frequently in the first post-operative year, and diffuse baseline aneurysmal disease and persistently elevated inflammatory markers might be negative prognosticators. We urge practitioners to monitor aortitis patients diligently with imaging and to have a high index of suspicion for dissection when symptomatic. Larger studies are required to corroborate our findings and, ultimately, a trial to evaluate any benefit of medical therapy.

*Funding*: No specific funding was received from any funding bodies in the public, commercial or not-for-profit sectors to carry out the work described in this manuscript.

*Disclosure statement*: The authors have declared no conflicts of interest.
